# The use of non‐adult vertebral dimensions as indicators of growth disruption and non‐specific health stress in skeletal populations

**DOI:** 10.1002/ajpa.22770

**Published:** 2015-06-29

**Authors:** Sophie L. Newman, Rebecca L. Gowland

**Affiliations:** ^1^ Department of ArchaeologyDurham UniversityDurhamDH1 3LEUK

**Keywords:** Vertebrae, axial growth, post‐medieval, puberty, stature

## Abstract

**Objective:**

Traditional methods of detecting growth disruption have focused on deficiencies in the diaphyseal length of the long bones. This study proposes the implementation of vertebral measurements (body height and transverse diameter of the neural canal) from non‐adults (0–17 years) as a new methodology for the identification of growth disruption.

**Methods:**

Measurements of vertebral body height and transverse diameter were taken from 96 non‐adult skeletons and 40 adult skeletons from two post‐medieval sites in England (Bow Baptist, London and Coronation Street, South Shields). Non‐adult measurements were plotted against dental age to construct vertebral growth profiles through which inter‐population comparisons could be made.

**Results:**

Results demonstrated that both sites experienced some growth retardation in infancy, evident as deficiencies in transverse diameter. However, analysis of vertebral body height revealed different chronologies of growth disruption between the sites, with a later age of attainment of skeletal maturity recorded in the Bow Baptist sample.

**Discussion:**

These vertebral dimensions undergo cessation of growth at different ages, with transverse diameter being “locked‐in” by ∼1–2 years of age, while vertebral body height may continue to grow into early adulthood. These measurements can therefore provide complementary information regarding the timing of growth disruption within archaeological populations. Non‐adult vertebral measurements can increase our osteobiographical understanding of the timings of episodes of health stress, and allow for the analysis of growth when other skeletal elements are fragmentary. Am J Phys Anthropol 158:155–164, 2015. © 2015 Wiley Periodicals, Inc.

The detrimental impact of chronic nutritional and health stress on the growth of children has long been recognized, and consequently adult stature and non‐adult growth are considered to be robust indicators of population health (Eveleth and Tanner, 1990; Saunders and Hoppa, 1993; Larsen, 1997; Lewis, 2007). The use of multiple indicators of stress has been an integral feature within bioarchaeology for many years (e.g., Goodman et al., 1984). However, a recent call to revitalize the way in which we interpret “health” and “stress” suggests that we should seek to implement a more comprehensive approach, and potentially pursue new avenues of research (Klaus, 2014; Temple and Goodman, 2014). While previous growth studies have relied on long bone length, more recently, other skeletal parameters such as cortical thickness, or compact bone geometry and histology, have provided fruitful new avenues for investigation (Mays et al., 2009; Robbins and Goldman, 2014). This study aims to add to this developing corpus by introducing non‐adult vertebral dimensions as a new method of detecting growth disruption in past populations.

## RESEARCH RATIONALE

The importance of the vertebrae in terms of non‐adult growth is best demonstrated by the renewed interest in the “anatomical” method of stature reconstruction from skeletal remains. This method does not rely on calculating stature from a single long bone using regression formulae (the “mathematical method”), but instead produces an estimate based upon the measurement of all of those bones that contribute to height (Raxter et al., 2006, 2007; Maijanen, 2009; Auerbach and Ruff, 2010; Auerbach, 2011; Vercellotti et al., 2014). This provides a more accurate method of calculating living stature, as it is not biased by differences in bodily proportions (Maijanen, 2009; Auerbach and Ruff, 2010; Vercellotti et al., 2014). As studies of stature are increasingly beginning to incorporate vertebral components, it is also now prudent to consider the growth of these skeletal elements.

## THE PROCESS OF VERTEBRAL GROWTH

Vertebral growth, while complex due to development around the spinal cord, shares similarities with that of long bone growth, in that both endochondral and intramembranous ossification occurs in order to create the adult form (Brandner, 1970; Reichmann and Lewin, 1971; Bogduk, 2005). Each vertebral component is formed from three primary centers of ossification, the centra (the body) and two halves of the neural arch (Scheuer and Black, 2000). It is the increase in vertebral body height and the development of the neural canal that is of interest to this study.

**Figure 1:**
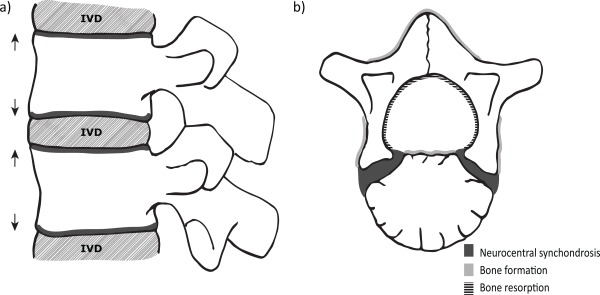
The process of vertebral growth. (**a**) Process of longitudinal vertebral body growth, depicting the growth plates (in dark gray) superior and inferior to the vertebral body, and the direction of growth (arrows). IVD = intervertebral disc. (**b**) Process of neural arch growth in a developing vertebra, with the neuro‐central synchondrosis (dark gray) and areas of bone formation (light gray) and resorption (black lines). (Adapted from Bogduk, 2005; Reichmann and Lewin, 1971).

An increase in vertebral body height is achieved via the process of columnar proliferation, differentiation, and mineralization of chondroblasts on the superior and inferior faces of each of the centra (Stevens and Williams, 1999; Wang et al., 2007). As new bone is laid down on these surfaces the vertebral column increases in length, therefore contributing to an increase in sitting height (Fig. 1a). This process displays three stages of growth throughout childhood and adolescence. There is a rapid increase in height from birth to 5 years of age, a period of quiescence between 5 and 10 years, and finally a pubertal growth spurt between ∼10.5 and 13.5 years of age in girls and 12.5 and 15.5 years in boys (Hefti and McMaster, 1983; Diméglio and Canavese, 2012). The formation of vertebral end plates (subchondral bone plates, not to be confused with the annular rings) at both the superior and inferior surfaces of the vertebral body marks the end of the growth period between 18 and 25 years of age (Bogduk, 2005).

While longitudinal growth of the vertebral body progresses throughout the growth period of an individual until maturity, the majority of neural arch growth is completed relatively early in postnatal growth. Cartilaginous growth plates that lie between the two halves of the neural arch (the spinous process), and between the neural arches and the centrum (the neuro‐central synchondroses), allow for the continued growth of the vertebral foramen around the developing spinal cord. This occurs via intramembranous bone formation and complementary bone resorption on the inner and outer surfaces of the neural canal (Fig. 1b) (Roaf, 1960; Reichmann and Lewin, 1971; Rajwani et al., 2002; Chen et al., 2006). Fusion of the spinous process occurs at ∼1–2 years of age (Jinkins, 2000; Scheuer and Black, 2000). The completed neural arch then fuses to the vertebral body at ∼3–5 years of age, completing the neural canal (Jinkins, 2000; Scheuer and Black, 2000). The formation of the anteroposterior (AP) and transverse diameters (TR) of the neural canal is predominantly complete in early childhood, reaching ∼95% of its final size by 5 years of age (Diméglio, 1993). Consequently, evidence of growth disturbance during early postnatal life may become “locked into” these dimensions (Clark et al., 1986; Clark, 1988; Diméglio, 1993; Larsen, 1997; Watts, 2011, 2013a,b). The value of the AP and TR dimensions of the vertebrae has been previously recognized and implemented in both adult (Clark et al., 1986; Clark, 1988; Watts, 2011, 2013a,b) and non‐adult (Watts, 2013b) skeletal collections. However, these measurements, as well as that of vertebral body height, have yet to be explored as a potential method to map the growth of non‐adults. Evidence suggests that vertebral growth proceeds in a manner analogous to long bone growth (Bick and Copel, 1950); therefore it is reasonable to expect disruptions to the development of both these elements under conditions of environmental stress. This study will assess the feasibility of the construction of vertebral growth profiles to detect episodes of “stress” within skeletal samples.

## MATERIALS AND METHODS

Two skeletal samples were selected for analysis so that the inter‐population comparability of the vertebral growth profiles could be assessed. The Bow Baptist skeletal collection of Payne Road, London represents a relatively prosperous post‐medieval population (c. AD 1816–1856) of 416 individuals, 202 of which are classed as “non‐adult” (0–17 years of age). The small village of Bow once existed as a separate entity to London, located on the eastern outskirts of the city. However, the second half of the 19th century saw a rapid industrialization of this area and its eventual incorporation into the expanding metropolis (Henderson et al., 2013).

The skeletal collection from Coronation Street, South Shields (c. AD 1816–1855), consists of 204 individuals, 90 of which are non‐adults. The site is located south of Newcastle‐upon‐Tyne and during this period was centered on local industries such as shipyards and collieries (Raynor et al., 2011). The individuals buried here are generally regarded as representative of a working‐class population.

Individuals between 0 and 17 years of age were selected from each collection, based on the presence of vertebrae whose position within the column could be reliably determined. Due to the ambiguous nature of vertebral body morphology in those aged less than one year, accurate identification was reliant on the majority of the vertebral elements being present. Any vertebrae that demonstrated signs of pathology (e.g., Schmorl's nodes) were omitted from this study, as were individuals with border shifts (when vertebrae develop features associated with the neighboring section, e.g. lumbarization of T12). These were removed as it is yet to be established whether these anomalies would have affected the course of vertebral development (Barnes, 1994; Watts 2011, 2013a,b).

In individuals where the necessary vertebral elements were present (68 in the Bow Baptist sample, and 28 in the Coronation Street sample) dental age was assessed using standards for dental formation of the deciduous and permanent dentition (Smith, 1991). Tooth formation stages were determined by examination of the dentition radiographically, or macroscopically when loose teeth were present (Pinhasi et al., 2005). These stages were used to assign a dental age to each individual, based on the mid‐point of the relevant age‐category. For example, those between 0.5 and 1.49 years of age were classed as 1 year of age, those between 1.5 and 2.45 as 2 years of age, and so on until 17 years of age. This methodology was chosen to ensure comparability of the data with previous growth studies (Mays et al., 2008). Individuals with no dentition preserved were omitted from the study.

### Vertebral measurements

**Figure 2:**
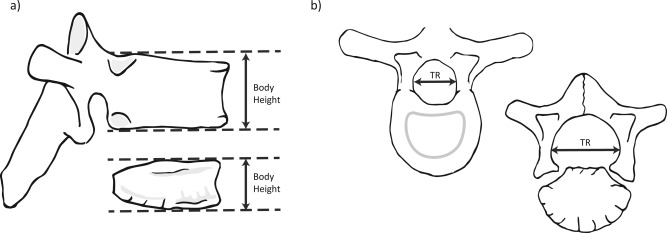
Measurements of vertebral growth. (**a**) Measurement points of vertebral body height in fused and unfused vertebrae. Taken from the midline of the centrum. (**b**) Measurement location of the transverse diameter of the neural canal in fused and unfused vertebrae.

Measurements of vertebral body height were taken from the midline of each centra (C3‐L5) at the point of maximum height. To achieve this, the inferior surface of the body was positioned horizontally on the sliding calipers and the mobile component moved until it touched the superior surface, thereby marking the point of maximum body height (Fig. 2). Measurements were taken to the nearest 0.01 mm.

**Figure 3:**
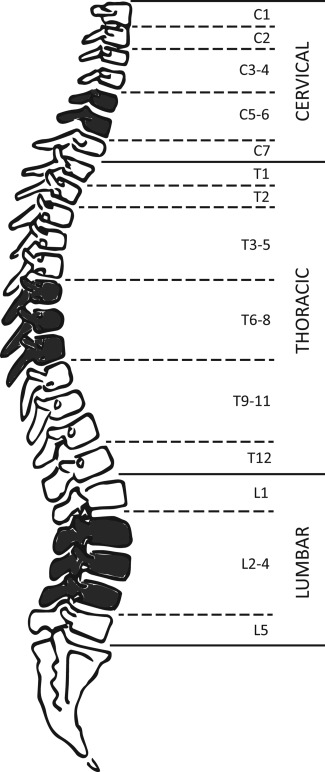
Vertebral groupings used for analysis of measurements. Shaded elements reflect the vertebral groups for which data will be presented within this study.

To assess vertebral neural canal size (VNC), measurements of TR diameter of the neural canal of cervical, thoracic, and lumbar vertebrae (C1‐L5) were taken using sliding calipers (to the nearest 0.01 mm). These measurements represented the furthest distance between the medial surfaces of the left and right pedicles (Watts, 2011, 2013a,b) and could only be taken when the neural arches had fused at the spinous process, though fusion of the neural arch to the vertebral body was not essential (Fig. 3). The AP diameter (normally taken from the posterior surface of the vertebral body to the furthest opposite point of the neural canal, anterior to the spinous process) (Watts, 2011, 2013a,b) was excluded from this study as these measurements could only be taken in individuals that had begun fusion of the neurocentral synchondrosis. Although this measurement is of great value in adult individuals, its use within non‐adult samples is restricted to those individuals whose neural arches and vertebral bodies have fused (i.e., older than approximately five years of age). This limitation means that sample sizes for AP diameter tend to be small, as older children and adolescents are often less common in skeletal collections (as can be seen in Table 2). Measurements of body height and TR diameter were also taken from adults aged 18–35 years in both collections to provide comparative data. A sample of 20 adults (10 males and 10 females) was selected from each site to enable comparability with the mixed‐sex non‐adult samples (see Table 1 for overall sample sizes for each measurement).

**Table 1:** Sample sizes for number of individuals from which measurements of body height (BH) and transverse diameter (TR) were taken. Adult sample sizes in brackets

	C5‐6		T6‐8		L2‐4	
Sites	BH	TR	BH	TR	BH	TR
Bow Baptist	49 (19)	42 (18)	42 (17)	46 (19)	40 (17)	43 (19)
Coronation Street	20 (11)	10 (15)	20 (11)	10 (13)	19 (10)	10 (14)

**Table 2:** Sample sizes for each non‐adult measurement, broken down by age category

	Coronation Street						Bow Baptist					
	C5‐6		T6‐8		L2‐4		C5‐6		T6‐8		L2‐4	
Age group	BH	TR	BH	TR	BH	TR	BH	TR	BH	TR	BH	TR
0	9	–	11	–	9	1	2	–	–	–	–	–
1	–	–	–	–	–	–	8	5	7	5	6	4
2	5	4	2	3	2	2	12	9	12	12	11	10
3	1	1	1	1	1	–	2	2	2	4	4	4
4	–	–	–	–	–	–	3	3	3	3	2	3
5	1	1	1	1	1	1	4	4	4	3	3	2
6	1	1	1	1	1	1	2	2	1	2	–	2
7	–	–	–	–	–	–	2	3	3	4	4	4
8	–	–	–	–	–	–	–	–	–	–	–	–
9	1	1	1	1	1	1	1	1	1	1	1	1
10	–	–	–	–	–	–	3	3	2	3	2	3
11	1	1	–	1	1	1	4	4	3	4	3	4
12	–	–	1	1	1	1	1	1	1	1	1	1
13	–	–	–	–	–	–	–	–	–	–	–	–
14	–	–	–	–	–	–	–	–	–	–	–	–
15	–	–	1	–	1	1	3	3	1	2	1	2
16	1	1	1	1	1	1	1	1	1	1	1	2
17	–	–	–	–	–	–	1	1	1	1	1	1
Total	20	10	20	10	19	10	49	42	42	46	40	43

**Figure 4:**
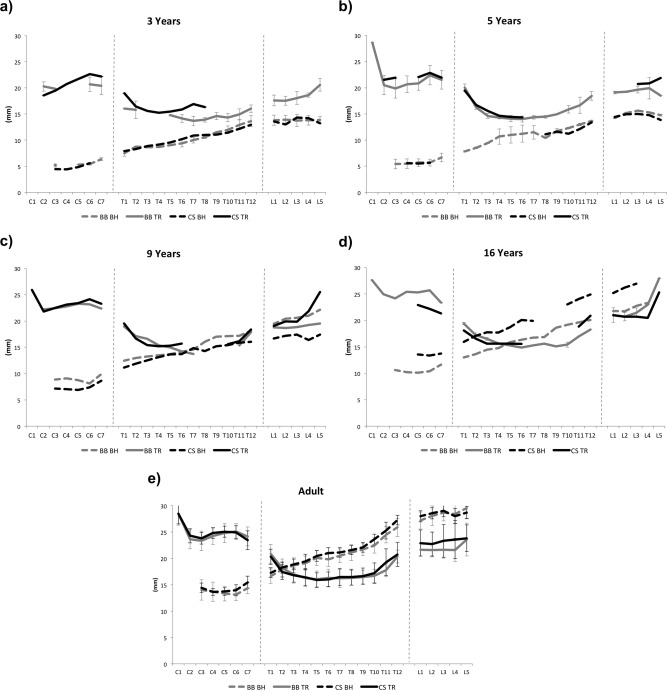
Summary graphs for the overall growth in body height and transverse diameter throughout the vertebral column at ages (**a**) 3, (**b**) 5, (**c**) 9, (**d**) 16, and (**e**) adult. BB = Bow Baptist; CS = Coronation Street.

Due to the linear increase in vertebral body height with age, it was possible to calculate measurements for missing vertebrae using the average value from the two adjacent vertebrae, when present (Auerbach, 2011). Once measurements had been taken, vertebrae were categorized into groups to maximize sample size (Fig. 3). The groups were delimited based on similarities in morphology of vertebrae, and transitional vertebrae (C7, T1, T12, L1, and L5) were considered separately. Averages of the vertebral measurements were taken for each age category and then plotted (C1‐L5) for ages 3, 5, 9, and 16 years to allow for a primary assessment of growth in the vertebral column (Fig. 4). These ages were chosen as they had the best representation of vertebral elements present in both samples throughout the growth period. Vertebral groups C5‐6, T6‐8, L2‐4 were chosen for further analysis due to their higher rate of preservation, and therefore larger sample sizes. The overall sample sizes for each vertebral group and measurement for both the non‐adult and adults within the two samples can be seen in Table 1, and the breakdown of sample sizes by age category for the non‐adult measurements in Table 2.

These grouped measurements were then plotted on scatter graphs against dental age to form vertebral growth profiles for C5‐6, T6‐8, and L2‐4.

The results for body height and TR diameter in these vertebral groups were statistically assessed via analysis of covariance (ANCOVA) to detect any potential differences in vertebral growth between the two samples. ANCOVA allows for the detection of differences between the regression slopes of two datasets, while acknowledging the influence of dental age as a covariate (Pinhasi et al., 2006). This method was only applied to individuals between 0 and 12 years of age to avoid the complications of the sex‐differentiated pubertal growth spurt, and to allow for comparison with the data from a modern sample for TR diameter (see below). An ANOVA test was also applied to the vertebral groups for the adult measurements of body height and TR diameter to establish whether any inter‐population differences identified in the non‐adult samples continued through to the end of the growth period.

## RESULTS

Figure 4 demonstrates the average size of each region in the vertebral column at ages 3, 5, 9, and 16 years, compared to the average adult measurements for each dimension. During infancy and early childhood, body height is similar between the Coronation Street and the Bow Baptist individuals (Fig. 4a,b). By 9 years of age differences in growth between the samples emerge, with Coronation Street demonstrating deficiencies in growth of the cervical, lumbar, and upper and lower thoracic vertebrae compared to the Bow Baptist individuals (Fig. 4c). This is most apparent in the lumbar portion of the column.

By 16 years of age the Coronation Street sample have, on average, attained 93% of the final adult body height throughout the vertebral column, while the Bow Baptist sample have only reached an average of 79% (Fig. 4d,e). The adult measurements themselves do not differ markedly, with the Bow Baptists measurements only slightly lower than those from Coronation Street between C2 and C4 and from T9 to L4 (Fig. 4e).

For the measurements of TR diameter, the non‐adult and adult values were similar, as expected due to the early age of fusion of this element (Fig. 4a–e). By 3 years of age, the non‐adults of the Bow Baptist and Coronation Street samples had attained ∼85% and 91% of the adult TR diameter respectively throughout the column. In contrast to the growth in body height, the TR diameter measurements do not vary greatly either within or between the skeletal samples. Likewise, the adult TR diameter measurements were similar between the two samples (Fig. 4e).

**Figure 5:**
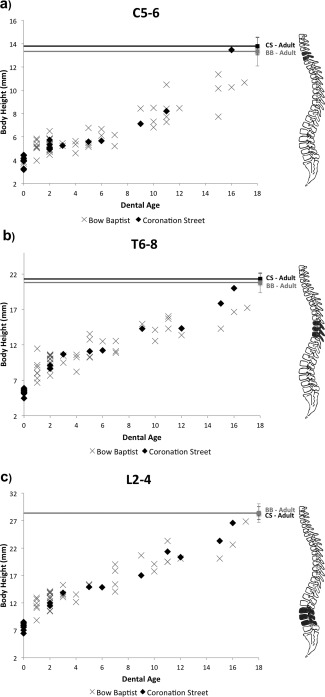
Vertebral growth profiles for body height for grouped vertebrae (**a**) C5‐6; (**b**) T6‐8; (**c**) L2‐4. Averages of adult body height for these groups shown as solid lines at the top of each graph.

For vertebral groups C5‐6, T6‐8, and L2‐4, measurements of body height and TR diameter were plotted against dental age as scatter graphs to form growth profiles. The growth profiles for body height also include data for the average body heights of the relevant adult vertebrae from each site. Figure 5 demonstrates that these data form a useable growth profile. Modern comparative data on vertebral body height is not currently available. Values for both the Bow Baptist and Coronation Street samples show statistically significant differences in the T6‐8 and L2‐4 vertebral groups (Table 4), with Coronation Street reaching on average only 42% of the adult sample average at 2 years of age for T6‐8, and 60% at 9 years of age for L2‐4. This is compared to 47% and 73% in the Bow Baptist population respectively. However, while the Coronation Street sample reaches the target adult proportions in all vertebral sections (Fig. 5a–c), this is only achieved by the Bow Baptist sample in L2‐4 (Fig. 5c). The individual of 17 years of age reached only 80% of the average adult body height for C5‐6, and ∼83% for T6‐8. Whereas an individual of 16 years of age from Coronation Street achieved values of ∼98% and 94% of the adult sample average. There were no statistically significant differences between the adult body heights of the Bow Baptist and Coronation Street samples (see Table 3).

**Table 3:** ANOVA results for adult measurements of body height (BH) and transverse diameter (TR) from the Coronation Street and Bow Baptist samples (P=<0.05)

	BH		TR	
	*F*	*P*	*F*	*P*
C5‐6	1.093	0.305	0.073	0.789
T6‐8	1.029	0.320	0.070	0.793
L2‐4	0.003	0.959	2.267	0.142

**Table 4:** ANCOVA results for the Bow Baptist and Coronation Street populations, including comparison of both sites with the modern sample for measurement of transverse diameter (P= <0.05)

	Body height		Transverse diameter	
	*F*	*P*	*F*	*P*
C5‐6	2.938	0.092	0.462	0.501
T6‐8	16.189	0.000	3.690	0.061
L2‐4	16.746	0.000	0.019	0.892
Modern				
C5‐6	–	–	21.945	0.000
T6‐8	–	–	9.339	0.000
L2‐4	–	–	11.096	0.000

**Figure 6:**
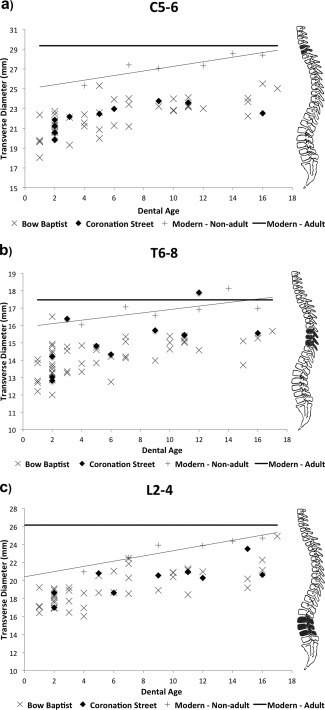
Vertebral growth profiles for transverse diameter for grouped vertebrae (**a**) C5‐6; (**b**) T6‐8; (**c**) L2‐4. Comparative modern adult data (solid black line) and modern non‐adult data (solid gray line) taken from Hinck et al. (1966).

Modern TR diameter data are available for comparison to the archaeological samples. The study by Hinck et al. (1966) provides average TR diameter measurements for 353 children up to 18 years of age (data averaged into ages 4, 7, 9, 12, 14, and 16), and also averages for measurements from 121 adults (classed as above 18 years of age). These data were plotted against the archaeological data‐sets (Fig. 6a–c). Both the modern data for children, and the archaeological non‐adult data, appear to have a slight upward trajectory. In the modern data, the trend line for the measurements of TR diameter in non‐adults meets that of the adult average value between 15 and 17 years of age.

The majority of individuals from both archaeological samples fall below modern values, most notably in the cervical and thoracic regions (Fig. 6a,b). This difference between the modern and archaeological samples was statistically significant for all three vertebral groups (Table 4). By 17 years of age, most individuals from the archaeological samples have not reached the expected measurements of TR diameter for a modern day healthy adult. While the Bow Baptist sample showed some of the lowest growth values in all three groups (Fig. 6a,c), there were no statistically significant differences in TR diameter between the two sites, unlike that seen for body height (Table 4).

## DISCUSSION

Earlier studies by Clark (Clark et al., 1986; Clark, 1988) and more recent studies by Watts (2011, 2013a,b) have successfully established the feasibility of detecting early episodes of stress within the AP and TR dimensions of the neural canal. However, techniques that incorporate vertebral dimensions into bioarchaeological research have yet to be fully explored in non‐adult skeletons. The most significant outcome of this study, therefore, is that it is possible to construct usable growth profiles from vertebral measurements, akin to those produced using long bone length.

The results of this study confirm an increase in vertebral body height throughout the entirety of the growth period, and an almost complete TR diameter by early childhood. It is of note, however, that there remains a minor increase in TR diameter (presumably due to remodeling of the neural canal) until ∼15–17 years of age. For example, at 2 years of age the Coronation Street and Bow Baptist samples have reached ∼81% and 86% of their adult sample values within the vertebral group T6‐8, respectively. By 16 years of age these values have both increased to 94%. This is indicative of a continuation of the growth period beyond the point of fusion of the vertebral arch to the vertebral body, contrary to the prior assumption that this measurement is completely “locked in” in infancy. While this result suggests a continuation in growth, it is also possible that the older children have larger TR diameters because they did not experience high levels of stress in infancy, thus represent “survivors.” Whereas the younger individuals have smaller diameters as they were “non‐survivors” and experienced heightened levels of stress in infancy, leading to an early age‐at‐death. The continuation of growth in the TR diameter of the neural canal has been noted by previous authors (Hinck et al., 1966; Reichmann and Lewin, 1971) and most recently by Watts (2013b). Nevertheless, the potential for catch‐up growth in this structure is still limited and the increase in size is relatively small. Therefore, it is still reasonable to assert that measurements of neural canal size are “locked in” in early childhood and consequently deficiencies can represent early life episodes of stress. Growth in the lumbar portion of the vertebral column demonstrates the greatest variability in TR diameter when compared to other regions (Fig. 4e). This requires further analysis to elucidate the cause of such variability.

The Bow Baptist site shows potential growth retardation in body height at 16 years of age compared to the Coronation Street sample, yet still demonstrates similar adult average body height values. Body height within the vertebral column can continue to grow between 18 and 25 years of age (Bogduk, 2005), therefore this suggests that body height may also undergo catch‐up growth and/or extended growth periods into early adulthood, analogous to that seen in long bone growth. This will need to be substantiated by further study due to the small sample size of the adolescent age group (Table 2). However, it does have implications for the study of stature and body proportions in past populations.

Both archaeological samples demonstrated deficiencies in TR diameter, with measurements falling significantly below that of modern “healthy” individuals. Some individuals within each data set are exceptionally underdeveloped, with the Bow Baptist individuals showing some of the lowest TR values. With regards to vertebral body height, comparison between the two archaeological samples reveals that the Bow Baptist non‐adults lag in growth between 9 and 16 years of age, and Coronation Street between 5 and 9 years of age (appearing to “recover” by 16 years of age when they are nearing/have met the average adult measurement values).

Crude prevalence rates (CPR) for other metabolic and non‐specific indicators of stress within each site were assessed to further elucidate these chronological patterns of vertebral growth disruption (see Table 5). Both the Bow Baptist and Coronation Street samples show very similar frequencies of dental enamel hypoplasia (DEH) (see Table 5). As tooth enamel of the deciduous and permanent dentition develops from the second trimester up until ∼10 years of age, these rates reflect non‐specific episodes of stress in infancy and childhood (Goodman and Rose, 1990). This evidence corroborates the deficiencies seen in the TR diameters of both archaeological populations. Overall crude prevalence rates for rickets, non‐specific infection (NSI), and cribra orbitalia within the non‐adult sample from Coronation Street were lower than the Bow Baptist sample (Table 5). These indicators of poor health correlate with the greater degree of growth disruption recorded in vertebral body height in the Bow Baptist sample.

**Table 5:** Crude prevalence rates (CPR) of skeletal indicators of stress in the Bow Baptist and Coronation non‐adult samples (Bow Baptist N=202, Coronation Street N=90)

	Bow Baptist	Coronation Street
DEH (%)	13.4	13.3
Rickets (%)	16.83	4.4
NSI (%)	11.88	4.4
Cribra Orbitalia (%)	13.37	10

The Bow Baptist population was of a higher social status when compared to the Coronation Street population. However, while the latter were regarded as “working class,” the wages paid in many of the local industries of South Shields would have provided sufficient means to buy food and shelter (Raynor et al., 2011). While child labor was common, the children of Coronation Street were more likely to have been employed in industries that involved outside work, such as ship‐building (Raynor et al., 2011). Dietary differences in the North and South also existed and historical evidence suggests that London diets were particularly poor, with foodstuffs often subjected to adulteration (Horrell and Oxley, 2012). Therefore, the growth deficiencies recorded in both body height and TR within the Bow Baptist sample from London, together with the higher prevalence of skeletal pathologies, may be related to exposure to harsher environmental stressors in London, which affected the middle as well as the poorer classes, during this period of industrialization.

This study highlights the potential of a combined approach to the measurement of both vertebral body height and TR diameter in non‐adults (alongside the use of existing indicators of non‐specific stress) to reveal further insight into the growth of individuals within skeletal samples. This combination of parameters allows growth to be analyzed during different life course stages, thus providing complementary osteobiographical data. The early childhood “lock in” in TR diameter means that the masking effect of catch‐up growth on this parameter is relatively minimal. These data have shown that while both of the archaeological samples faced episodes of stress in infancy and early childhood, the timing of later episodes of growth disruption differed. This suggests different timings of vulnerability throughout the growth period between the two sites, due to differences in environmental risk and/or age‐related cultural practices.

The differences between sites in terms of attainment of final vertebral body height highlight the potential for catch‐up growth within the vertebral column. Final height is produced from both the appendicular and axial skeleton and therefore primarily depends on the differential timings of growth acceleration before and after puberty. In the pre‐pubertal stage of adolescence, appendicular growth is more rapid than axial growth. In early puberty, while appendicular growth maintains a more constant rate of growth, axial growth undergoes acceleration (Bass et al., 1999; Bradney et al., 2000; Seeman et al., 2000). Through this tempo of growth, the bones of the limbs would be expected to reach their final size at an earlier age than those of the vertebral column (Nyati et al., 2006). Therefore, depending on the age of exposure to stress, the resulting growth disruption may have a differential effect on the developing skeletal elements, with those undergoing fastest growth being most affected (Bass et al., 1999). Shortly before puberty, growth disruption is most likely to be evident in the limbs; therefore the long bones would be expected to show deficiencies in length (Bass et al., 1999; Bradney et al., 2000). Whereas during puberty the axial skeleton is more likely to be deviated from its developmental trajectory and vertebral body height is more likely to demonstrate stunted growth (Riggs et al., 1999; Bradney et al., 2000).

This has interesting applications to the study of the growth period and stature in bioarchaeology. The combination of both longitudinal growth and vertebral growth may prove to be fruitful in terms of better understanding episodes of stress during the growth period. However, one current limitation is that the relationship between puberty, growth, and epiphyseal fusion is highly differentiated by sex. Girls undergo the onset of puberty, and therefore pubertal growth spurts, ∼2 years earlier than males (girls ∼10–11 years, and boys ∼11–13 years, peaking at 12 and 14 years respectively) (Tanner, 1989; Diméglio, 2001). The onset of puberty, however, is influenced by environmental conditions, nutritional status, and episodes of disease (Shapland and Lewis, 2013). Historical evidence from 19th century England alludes to the impact of environment (in particular child labor conditions) on pubertal timing, referring to cases of both premature and delayed puberty in young factory workers (Engels, 1971).

Sexual dimorphism has long been recognized as a limitation in past studies of long bone growth. Male vertebral dimensions have been found to be larger than females during both adolescence and adulthood (Taylor and Twomey, 1984; Gilsanz et al., 1994, 1997; Bastir et al., 2014). However, Gilsanz et al. (1994, 1997) found that while the cross‐sectional area of the vertebral bodies was larger in boys than girls, measurements of vertebral body height were comparable at all ages (Gilsanz et al., 1994, 1997). Limited sexual dimorphism in the TR diameters of adult males and females has also been reported and this is an important advantage of studies of vertebral growth (Clark et al., 1986; Watts, 2011). However, advancement in the ability to assess pubertal development provides an additional avenue through which vertebral growth can be assessed in the future (Shapland and Lewis, 2013).

All bioarchaeological studies of non‐adult growth must contend with preservation problems and small sample sizes. As can be seen in Table 2, many of the age categories are represented by only one individual, particularly within Coronation Street. Therefore it must be acknowledged that the results of this study are based on a very limited sample. Furthermore, the cross‐sectional nature of archaeological data‐sets and the use of dental age as a proxy for known age will introduce additional potential sources of bias. Some of these biases can be mitigated with the application of appropriate statistical techniques and the adoption of suitable caution when interpreting findings. Overall, however, the use of vertebral growth parameters has the potential to enrich our knowledge of growth in the past within a variety of social and environmental contexts.

## CONCLUSION

This study has sought to introduce and evaluate the potential of vertebral parameters for the bioarchaeological examination of skeletal remains. It has demonstrated that both vertebral body height and the TR diameter of the neural canal have the potential to provide a commentary on growth lag, catch‐up growth, and early life experiences in past populations. These data were also corroborated by the prevalence of skeletal pathologies within these two samples, indicating that this method can be used alongside existing indicators of stress. This is significant for providing osteobiographical data concerning the likely age at which stress events occurred. Further application to other bioarchaeological collections is now required in order to increase data‐sets and to examine growth in relation to other non‐specific skeletal indicators of poor health. The vertebral measurements discussed above provide another means by which we can access the growth profile of past children, whether in conjunction with other growth parameters, such as long bone length, or as a substitute when long bones are fragmentary.
